# Supplementary material for: Pyruvate Dehydrogenase Kinase 1 inhibition mediated oxidative phosphorylation enhancement in cartilage promotes osteoarthritis progression
Source: BMC Musculoskelet Disord. 2023 Jul 20;24:597. doi: 10.1186/s12891-023-06585-6 (PMC10357736; doi:10.1186/s12891-023-06585-6)
Supplement: Supplementary file 1 — Additional file 1. [file 12891_2023_6585_MOESM1_ESM.zip › BP.Gsea.1653623667859/GOBP_PROTON_TRANSMEMBRANE_TRANSPORT.html]

Details for gene set GOBP\_PROTON\_TRANSMEMBRANE\_TRANSPORT[GSEA]

|  || Dataset | 55457\_collapsed\_to\_symbols.cls.cls #OA\_versus\_normal.cls.cls #OA\_versus\_normal\_repos |
| Phenotype | cls.cls#OA\_versus\_normal\_repos |
| Upregulated in class | OA |
| GeneSet | GOBP\_PROTON\_TRANSMEMBRANE\_TRANSPORT |
| Enrichment Score (ES) | 0.45400834 |
| Normalized Enrichment Score (NES) | 2.1372757 |
| Nominal p-value | 0.0 |
| FDR q-value | 0.0048259865 |
| FWER p-Value | 0.077 |
Table: GSEA Results Summary

  

Fig 1: Enrichment plot: GOBP\_PROTON\_TRANSMEMBRANE\_TRANSPORT      
 Profile of the Running ES Score & Positions of GeneSet Members on the Rank Ordered List

  

| SYMBOL | TITLE | RANK IN GENE LIST | RANK METRIC SCORE | RUNNING ES | CORE ENRICHMENT || 1 | TMCO3 | transmembrane and coiled-coil domains 3 [Source:HGNC Symbol;Acc:HGNC:20329] | 12 | 1.165 | 0.0287 | Yes |
| 2 | ATP6V1C1 | ATPase H+ transporting V1 subunit C1 [Source:HGNC Symbol;Acc:HGNC:856] | 17 | 1.125 | 0.0570 | Yes |
| 3 | UQCR10 | "ubiquinol-cytochrome c reductase, complex III subunit X [Source:HGNC Symbol;Acc:HGNC:30863]" | 53 | 0.987 | 0.0794 | Yes |
| 4 | COX17 | cytochrome c oxidase copper chaperone COX17 [Source:HGNC Symbol;Acc:HGNC:2264] | 64 | 0.966 | 0.1032 | Yes |
| 5 | ATP6V0D1 | ATPase H+ transporting V0 subunit d1 [Source:HGNC Symbol;Acc:HGNC:13724] | 135 | 0.872 | 0.1200 | Yes |
| 6 | SLC22A18 | solute carrier family 22 member 18 [Source:HGNC Symbol;Acc:HGNC:10964] | 152 | 0.858 | 0.1406 | Yes |
| 7 | ATP6V1E1 | ATPase H+ transporting V1 subunit E1 [Source:HGNC Symbol;Acc:HGNC:857] | 287 | 0.758 | 0.1494 | Yes |
| 8 | ATP5MC1 | ATP synthase membrane subunit c locus 1 [Source:HGNC Symbol;Acc:HGNC:841] | 465 | 0.674 | 0.1527 | Yes |
| 9 | UQCRC1 | ubiquinol-cytochrome c reductase core protein 1 [Source:HGNC Symbol;Acc:HGNC:12585] | 473 | 0.672 | 0.1693 | Yes |
| 10 | ATP6V1G1 | ATPase H+ transporting V1 subunit G1 [Source:HGNC Symbol;Acc:HGNC:864] | 515 | 0.655 | 0.1828 | Yes |
| 11 | ATP6V0E2 | ATPase H+ transporting V0 subunit e2 [Source:HGNC Symbol;Acc:HGNC:21723] | 532 | 0.647 | 0.1980 | Yes |
| 12 | ATP5PD | ATP synthase peripheral stalk subunit d [Source:HGNC Symbol;Acc:HGNC:845] | 564 | 0.635 | 0.2117 | Yes |
| 13 | COX5B | cytochrome c oxidase subunit 5B [Source:HGNC Symbol;Acc:HGNC:2269] | 585 | 0.628 | 0.2262 | Yes |
| 14 | ANTKMT | adenine nucleotide translocase lysine methyltransferase [Source:HGNC Symbol;Acc:HGNC:14152] | 618 | 0.618 | 0.2394 | Yes |
| 15 | NDUFA4 | NDUFA4 mitochondrial complex associated [Source:HGNC Symbol;Acc:HGNC:7687] | 624 | 0.613 | 0.2546 | Yes |
| 16 | COX6A1 | cytochrome c oxidase subunit 6A1 [Source:HGNC Symbol;Acc:HGNC:2277] | 653 | 0.605 | 0.2678 | Yes |
| 17 | DMAC2L | distal membrane arm assembly component 2 like [Source:HGNC Symbol;Acc:HGNC:18799] | 695 | 0.595 | 0.2798 | Yes |
| 18 | ATP5PO | ATP synthase peripheral stalk subunit OSCP [Source:HGNC Symbol;Acc:HGNC:850] | 840 | 0.558 | 0.2827 | Yes |
| 19 | SLC2A10 | solute carrier family 2 member 10 [Source:HGNC Symbol;Acc:HGNC:13444] | 874 | 0.550 | 0.2941 | Yes |
| 20 | TACO1 | translational activator of cytochrome c oxidase I [Source:HGNC Symbol;Acc:HGNC:24316] | 923 | 0.536 | 0.3040 | Yes |
| 21 | SLC9A6 | solute carrier family 9 member A6 [Source:HGNC Symbol;Acc:HGNC:11079] | 981 | 0.522 | 0.3129 | Yes |
| 22 | NDUFS7 | NADH:ubiquinone oxidoreductase core subunit S7 [Source:HGNC Symbol;Acc:HGNC:7714] | 1071 | 0.501 | 0.3187 | Yes |
| 23 | COX8A | cytochrome c oxidase subunit 8A [Source:HGNC Symbol;Acc:HGNC:2294] | 1105 | 0.494 | 0.3287 | Yes |
| 24 | ATP6V0E1 | ATPase H+ transporting V0 subunit e1 [Source:HGNC Symbol;Acc:HGNC:863] | 1207 | 0.474 | 0.3329 | Yes |
| 25 | UCP2 | uncoupling protein 2 [Source:HGNC Symbol;Acc:HGNC:12518] | 1270 | 0.464 | 0.3398 | Yes |
| 26 | ATP5PB | ATP synthase peripheral stalk-membrane subunit b [Source:HGNC Symbol;Acc:HGNC:840] | 1293 | 0.459 | 0.3498 | Yes |
| 27 | ATP6V1H | ATPase H+ transporting V1 subunit H [Source:HGNC Symbol;Acc:HGNC:18303] | 1304 | 0.457 | 0.3606 | Yes |
| 28 | ATP5MF | ATP synthase membrane subunit f [Source:HGNC Symbol;Acc:HGNC:848] | 1330 | 0.453 | 0.3702 | Yes |
| 29 | PARK7 | Parkinsonism associated deglycase [Source:HGNC Symbol;Acc:HGNC:16369] | 1406 | 0.442 | 0.3756 | Yes |
| 30 | CLCN3 | chloride voltage-gated channel 3 [Source:HGNC Symbol;Acc:HGNC:2021] | 1535 | 0.423 | 0.3764 | Yes |
| 31 | SLC2A9 | solute carrier family 2 member 9 [Source:HGNC Symbol;Acc:HGNC:13446] | 1634 | 0.408 | 0.3791 | Yes |
| 32 | STOML2 | stomatin like 2 [Source:HGNC Symbol;Acc:HGNC:14559] | 1819 | 0.382 | 0.3744 | Yes |
| 33 | SURF1 | SURF1 cytochrome c oxidase assembly factor [Source:HGNC Symbol;Acc:HGNC:11474] | 1829 | 0.381 | 0.3834 | Yes |
| 34 | SLC30A5 | solute carrier family 30 member 5 [Source:HGNC Symbol;Acc:HGNC:19089] | 1940 | 0.368 | 0.3842 | Yes |
| 35 | COX7A1 | cytochrome c oxidase subunit 7A1 [Source:HGNC Symbol;Acc:HGNC:2287] | 2045 | 0.356 | 0.3851 | Yes |
| 36 | SLC9A3 | solute carrier family 9 member A3 [Source:HGNC Symbol;Acc:HGNC:11073] | 2148 | 0.344 | 0.3859 | Yes |
| 37 | COX4I1 | cytochrome c oxidase subunit 4I1 [Source:HGNC Symbol;Acc:HGNC:2265] | 2167 | 0.342 | 0.3932 | Yes |
| 38 | ATP5F1C | ATP synthase F1 subunit gamma [Source:HGNC Symbol;Acc:HGNC:833] | 2190 | 0.339 | 0.4001 | Yes |
| 39 | ATP12A | ATPase H+/K+ transporting non-gastric alpha2 subunit [Source:HGNC Symbol;Acc:HGNC:13816] | 2330 | 0.323 | 0.3975 | Yes |
| 40 | UCP1 | uncoupling protein 1 [Source:HGNC Symbol;Acc:HGNC:12517] | 2339 | 0.322 | 0.4051 | Yes |
| 41 | ATP5MC2 | ATP synthase membrane subunit c locus 2 [Source:HGNC Symbol;Acc:HGNC:842] | 2371 | 0.318 | 0.4108 | Yes |
| 42 | NNT | nicotinamide nucleotide transhydrogenase [Source:HGNC Symbol;Acc:HGNC:7863] | 2425 | 0.311 | 0.4145 | Yes |
| 43 | SPHK2 | sphingosine kinase 2 [Source:HGNC Symbol;Acc:HGNC:18859] | 2478 | 0.305 | 0.4182 | Yes |
| 44 | CLCN4 | chloride voltage-gated channel 4 [Source:HGNC Symbol;Acc:HGNC:2022] | 2498 | 0.303 | 0.4244 | Yes |
| 45 | COX7A2L | cytochrome c oxidase subunit 7A2 like [Source:HGNC Symbol;Acc:HGNC:2289] | 2517 | 0.302 | 0.4307 | Yes |
| 46 | SLC15A3 | solute carrier family 15 member 3 [Source:HGNC Symbol;Acc:HGNC:18068] | 2612 | 0.290 | 0.4307 | Yes |
| 47 | ATP5MC3 | ATP synthase membrane subunit c locus 3 [Source:HGNC Symbol;Acc:HGNC:843] | 2643 | 0.286 | 0.4357 | Yes |
| 48 | SLC17A5 | solute carrier family 17 member 5 [Source:HGNC Symbol;Acc:HGNC:10933] | 2671 | 0.283 | 0.4408 | Yes |
| 49 | ATP5F1D | ATP synthase F1 subunit delta [Source:HGNC Symbol;Acc:HGNC:837] | 2701 | 0.279 | 0.4456 | Yes |
| 50 | ATP6V0C | ATPase H+ transporting V0 subunit c [Source:HGNC Symbol;Acc:HGNC:855] | 2790 | 0.271 | 0.4456 | Yes |
| 51 | SLC9A7 | solute carrier family 9 member A7 [Source:HGNC Symbol;Acc:HGNC:17123] | 2817 | 0.268 | 0.4504 | Yes |
| 52 | COX6B1 | cytochrome c oxidase subunit 6B1 [Source:HGNC Symbol;Acc:HGNC:2280] | 2915 | 0.259 | 0.4494 | Yes |
| 53 | ATP5MG | ATP synthase membrane subunit g [Source:HGNC Symbol;Acc:HGNC:14247] | 2941 | 0.256 | 0.4540 | Yes |
| 54 | ATP5F1B | ATP synthase F1 subunit beta [Source:HGNC Symbol;Acc:HGNC:830] | 3024 | 0.247 | 0.4539 | No |
| 55 | DRD4 | dopamine receptor D4 [Source:HGNC Symbol;Acc:HGNC:3025] | 3267 | 0.224 | 0.4407 | No |
| 56 | SLC33A1 | solute carrier family 33 member 1 [Source:HGNC Symbol;Acc:HGNC:95] | 3320 | 0.219 | 0.4422 | No |
| 57 | CTNS | "cystinosin, lysosomal cystine transporter [Source:HGNC Symbol;Acc:HGNC:2518]" | 3337 | 0.218 | 0.4465 | No |
| 58 | COX7B | cytochrome c oxidase subunit 7B [Source:HGNC Symbol;Acc:HGNC:2291] | 3452 | 0.207 | 0.4428 | No |
| 59 | ATP6V1G2 | ATPase H+ transporting V1 subunit G2 [Source:HGNC Symbol;Acc:HGNC:862] | 3709 | 0.184 | 0.4275 | No |
| 60 | COX15 | cytochrome c oxidase assembly homolog COX15 [Source:HGNC Symbol;Acc:HGNC:2263] | 3727 | 0.183 | 0.4309 | No |
| 61 | UQCRH | ubiquinol-cytochrome c reductase hinge protein [Source:HGNC Symbol;Acc:HGNC:12590] | 3827 | 0.174 | 0.4275 | No |
| 62 | ATP6V1F | ATPase H+ transporting V1 subunit F [Source:HGNC Symbol;Acc:HGNC:16832] | 3833 | 0.173 | 0.4316 | No |
| 63 | SLC9A3R1 | SLC9A3 regulator 1 [Source:HGNC Symbol;Acc:HGNC:11075] | 4111 | 0.145 | 0.4136 | No |
| 64 | ATP5F1A | ATP synthase F1 subunit alpha [Source:HGNC Symbol;Acc:HGNC:823] | 4137 | 0.143 | 0.4153 | No |
| 65 | SLC9A1 | solute carrier family 9 member A1 [Source:HGNC Symbol;Acc:HGNC:11071] | 4221 | 0.135 | 0.4122 | No |
| 66 | NOX5 | NADPH oxidase 5 [Source:HGNC Symbol;Acc:HGNC:14874] | 4263 | 0.131 | 0.4124 | No |
| 67 | PHB2 | prohibitin 2 [Source:HGNC Symbol;Acc:HGNC:30306] | 4354 | 0.124 | 0.4085 | No |
| 68 | CLCN5 | chloride voltage-gated channel 5 [Source:HGNC Symbol;Acc:HGNC:2023] | 4372 | 0.123 | 0.4103 | No |
| 69 | ATP7A | ATPase copper transporting alpha [Source:HGNC Symbol;Acc:HGNC:869] | 4814 | 0.086 | 0.3780 | No |
| 70 | CYC1 | cytochrome c1 [Source:HGNC Symbol;Acc:HGNC:2579] | 4879 | 0.082 | 0.3751 | No |
| 71 | SLC9A2 | solute carrier family 9 member A2 [Source:HGNC Symbol;Acc:HGNC:11072] | 5068 | 0.067 | 0.3621 | No |
| 72 | SLC25A4 | solute carrier family 25 member 4 [Source:HGNC Symbol;Acc:HGNC:10990] | 5073 | 0.067 | 0.3635 | No |
| 73 | COX6A2 | cytochrome c oxidase subunit 6A2 [Source:HGNC Symbol;Acc:HGNC:2279] | 5242 | 0.053 | 0.3517 | No |
| 74 | COX5A | cytochrome c oxidase subunit 5A [Source:HGNC Symbol;Acc:HGNC:2267] | 5329 | 0.045 | 0.3461 | No |
| 75 | ATP1A3 | ATPase Na+/K+ transporting subunit alpha 3 [Source:HGNC Symbol;Acc:HGNC:801] | 5475 | 0.035 | 0.3357 | No |
| 76 | UQCRFS1 | "ubiquinol-cytochrome c reductase, Rieske iron-sulfur polypeptide 1 [Source:HGNC Symbol;Acc:HGNC:12587]" | 5509 | 0.032 | 0.3339 | No |
| 77 | CYB5A | cytochrome b5 type A [Source:HGNC Symbol;Acc:HGNC:2570] | 5850 | 0.005 | 0.3074 | No |
| 78 | ATP6V0A4 | ATPase H+ transporting V0 subunit a4 [Source:HGNC Symbol;Acc:HGNC:866] | 5968 | -0.002 | 0.2984 | No |
| 79 | ATP5PF | ATP synthase peripheral stalk subunit F6 [Source:HGNC Symbol;Acc:HGNC:847] | 6109 | -0.013 | 0.2878 | No |
| 80 | ATP5ME | ATP synthase membrane subunit e [Source:HGNC Symbol;Acc:HGNC:846] | 6365 | -0.032 | 0.2686 | No |
| 81 | ATP5F1E | ATP synthase F1 subunit epsilon [Source:HGNC Symbol;Acc:HGNC:838] | 6405 | -0.036 | 0.2665 | No |
| 82 | SLC15A1 | solute carrier family 15 member 1 [Source:HGNC Symbol;Acc:HGNC:10920] | 6515 | -0.043 | 0.2591 | No |
| 83 | SLC15A2 | solute carrier family 15 member 2 [Source:HGNC Symbol;Acc:HGNC:10921] | 6704 | -0.056 | 0.2458 | No |
| 84 | ATP6V1B2 | ATPase H+ transporting V1 subunit B2 [Source:HGNC Symbol;Acc:HGNC:854] | 7074 | -0.086 | 0.2191 | No |
| 85 | DRD3 | dopamine receptor D3 [Source:HGNC Symbol;Acc:HGNC:3024] | 7101 | -0.088 | 0.2194 | No |
| 86 | TCIRG1 | "T cell immune regulator 1, ATPase H+ transporting V0 subunit a3 [Source:HGNC Symbol;Acc:HGNC:11647]" | 7600 | -0.127 | 0.1837 | No |
| 87 | SLC36A1 | solute carrier family 36 member 1 [Source:HGNC Symbol;Acc:HGNC:18761] | 7670 | -0.132 | 0.1816 | No |
| 88 | SLC25A5 | solute carrier family 25 member 5 [Source:HGNC Symbol;Acc:HGNC:10991] | 7877 | -0.146 | 0.1693 | No |
| 89 | ATP6V1A | ATPase H+ transporting V1 subunit A [Source:HGNC Symbol;Acc:HGNC:851] | 8013 | -0.156 | 0.1627 | No |
| 90 | ATP1A2 | ATPase Na+/K+ transporting subunit alpha 2 [Source:HGNC Symbol;Acc:HGNC:800] | 8218 | -0.173 | 0.1511 | No |
| 91 | ATP1A1 | ATPase Na+/K+ transporting subunit alpha 1 [Source:HGNC Symbol;Acc:HGNC:799] | 8282 | -0.177 | 0.1507 | No |
| 92 | SLC25A3 | solute carrier family 25 member 3 [Source:HGNC Symbol;Acc:HGNC:10989] | 8681 | -0.208 | 0.1249 | No |
| 93 | SLC25A22 | solute carrier family 25 member 22 [Source:HGNC Symbol;Acc:HGNC:19954] | 8682 | -0.208 | 0.1302 | No |
| 94 | SLC11A2 | solute carrier family 11 member 2 [Source:HGNC Symbol;Acc:HGNC:10908] | 9152 | -0.244 | 0.0997 | No |
| 95 | PPIF | peptidylprolyl isomerase F [Source:HGNC Symbol;Acc:HGNC:9259] | 9999 | -0.321 | 0.0418 | No |
| 96 | SLC9A8 | solute carrier family 9 member A8 [Source:HGNC Symbol;Acc:HGNC:20728] | 10170 | -0.338 | 0.0371 | No |
| 97 | ATP4A | ATPase H+/K+ transporting subunit alpha [Source:HGNC Symbol;Acc:HGNC:819] | 10417 | -0.363 | 0.0271 | No |
| 98 | SLC47A1 | solute carrier family 47 member 1 [Source:HGNC Symbol;Acc:HGNC:25588] | 10423 | -0.363 | 0.0359 | No |
| 99 | ACTN4 | actinin alpha 4 [Source:HGNC Symbol;Acc:HGNC:166] | 10472 | -0.370 | 0.0416 | No |
| 100 | UCP3 | uncoupling protein 3 [Source:HGNC Symbol;Acc:HGNC:12519] | 10565 | -0.380 | 0.0441 | No |
| 101 | TMSB4X | thymosin beta 4 X-linked [Source:HGNC Symbol;Acc:HGNC:11881] | 10741 | -0.398 | 0.0405 | No |
| 102 | CHP1 | calcineurin like EF-hand protein 1 [Source:HGNC Symbol;Acc:HGNC:17433] | 10940 | -0.423 | 0.0358 | No |
| 103 | VPS9D1 | VPS9 domain containing 1 [Source:HGNC Symbol;Acc:HGNC:13526] | 11495 | -0.501 | 0.0053 | No |
| 104 | TWIST1 | twist family bHLH transcription factor 1 [Source:HGNC Symbol;Acc:HGNC:12428] | 11696 | -0.531 | 0.0032 | No |
| 105 | TESC | tescalcin [Source:HGNC Symbol;Acc:HGNC:26065] | 11793 | -0.548 | 0.0096 | No |
| 106 | IL13 | interleukin 13 [Source:HGNC Symbol;Acc:HGNC:5973] | 12173 | -0.636 | -0.0038 | No |
| 107 | ATP6V1B1 | ATPase H+ transporting V1 subunit B1 [Source:HGNC Symbol;Acc:HGNC:853] | 12206 | -0.646 | 0.0101 | No |
| 108 | SLC9A5 | solute carrier family 9 member A5 [Source:HGNC Symbol;Acc:HGNC:11078] | 12642 | -0.820 | -0.0030 | No |
| 109 | MT-ND5 | mitochondrially encoded NADH:ubiquinone oxidoreductase core subunit 5 [Source:HGNC Symbol;Acc:HGNC:7461] | 12743 | -0.906 | 0.0123 | No |
Table: GSEA details [plain text format]

  

Fig 2: GOBP\_PROTON\_TRANSMEMBRANE\_TRANSPORT      
 Blue-Pink O' Gram in the Space of the Analyzed GeneSet

  

Fig 3: GOBP\_PROTON\_TRANSMEMBRANE\_TRANSPORT: Random ES distribution      
 Gene set null distribution of ES for **GOBP\_PROTON\_TRANSMEMBRANE\_TRANSPORT**
